# Therapeutic effect of imiquimod on dextran sulfate sodium-induced ulcerative colitis in mice

**DOI:** 10.1371/journal.pone.0186138

**Published:** 2017-10-19

**Authors:** Lu Chen, Zhongyin Zhou, Yan Yang, Na Chen, Hongyu Xiang

**Affiliations:** Department of Gastroenterology, Hubei Key Laboratory of Digestive System Disease, RenMin Hospital of Wuhan University, Wuhan, China; Cairo University Faculty of Pharmacy, EGYPT

## Abstract

**Background:**

Imiquimod is a Toll-like receptor-7 agonist that regulates immunity and can be used as an immune adjuvant. Ulcerative colitis has a close correlation with immune disorder.

**Aim:**

To investigate the therapeutic effect of imiquimod on dextran sulfate sodium (DSS)-induced colitis and explore the underlying mechanisms.

**Methods:**

C57BL/6J C57 mice received 3% DSS for 7 days to induce ulcerative colitis. Groups of mice were intraperitoneally injected with dexamethasone (DXM, 1.5 mg/kg) or imiquimod (IMQ, 30 mg/kg) at the same time daily. During the experimental period, clinical signs, body weight, stool consistency and visible fecal blood were monitored and recorded daily; colitis was evaluated by disease activity index (DAI) score and by histological score. At the conclusion of the experiment, the level of colonic myeloperoxidase (MPO) activity and the serum levels of the cytokines tumor necrosis factor-α (TNF-α), interleukin 6 (IL-6) and interleukin 10 (IL-10) were measured.

**Results:**

Administration of 3% DSS for 7 days successfully induced acute colitis associated with diarrhea, bloody mucopurulent stool, body weight decreases, and other changes. Colitis severity was significantly ameliorated in the IMQ treatment groups, as determined by hematoxylin-eosin (HE) staining and histopathological scores. Moreover, IMQ significantly reduced the activity of MPO in colonic tissue and the serum levels of inflammatory cytokines, increased colon length and spleen weight, and effectively inhibited microscopic damage to the colon tissue.

**Conclusion:**

IMQ had beneficial effects on DSS-induced ulcerative colitis, supporting its further development and clinical application in ulcerative colitis.

## Introduction

Ulcerative colitis (UC), whose etiology is not very clear yet, is a chronic, nonspecific inflammatory bowel disease (IBD) whose lesions are mainly limited to the sigmoid colon and the rectal mucosa and submucosa depending on the severity of disease, and it has a chronic and recurrent course [1]. On account of its unknown causes, recurrent trends, protracted course, and tendency to cause a variety of concerning parenteral symptoms, the World Health Organization has identified UC as one of the modern refractory diseases [2]. At present, its pathogenesis is not clearly understood, and it is generally considered to be caused by interactions among genetics, environment, infection, dysbacteriosis and some other factors [3, 4]. However, most scholars believe that the pathogenetic mechanisms of UC and immune disorders are directly connected. Immune disorders play a vital role in the by which UC occurs, develops and transforms [5]. The current hypothesis states that IBD results from an inappropriately elevated immune response to resident intestinal bacteria [6]. A great quantity of experiments demonstrated that if the levels of proinflammatory cytokines (e.g., TNF-α, IL-1β, IL-6) in a UC patient’s body increase or the level of the anti-inflammatory cytokine IL-10 decreases, there will be obvious chronic inflammation in the gut [7]. It has been shown that colonic homeostasis is maintained by the innate immune systems, mainly via Toll-like receptor (TLR) signaling [8, 9]. Therefore, counteracting immune disorders can improve the recovery rate, shorten the duration of symptoms, and reduce the complications of UC. This immune approach may offer a new direction for treatment.

IMQ is a type of non-nucleoside isoimidazole quinoline drug that is mainly used for the topical therapy of skin diseases, such as verruca acuminata, keratosis and HPV-related epithelial dysplasia [10–12]. It was recently found that IMQ is virus- and tumor-resistant and can be used as an activator and B-lymphocyte immune adjuvant [13] to change the manner of the immune response, induce a specific immune response and improve the body’s protective immunity. IMQ does not show direct activity against the viruses that cause warts [14]. It, leading to produce cytokines and chemokines in a similar manner to TLR9, appears to act by activating the local innate immune response through Toll-like 7 (TLR7) stimulation [15]. However, its precise molecular mechanism of action remains unclear [16]. Since it is currently not known whether IMQ is able to improve the immune disorders of UC patients, studying the curative effect and mechanism of IMQ to UC and viewing it as a bridge to connect UC with immune disorder will lead to an innovative approach to UC treatment. This study investigated the curative effect and mechanism of IMQ against UC by establishing animal models.

## Materials and methods

### Animals

Eight-week-old wild-type male C57BL/6J mice were purchased from Vital River Laboratory Animal Technology Co., Ltd. (certificate No. 32002100001086). Prior to the experiment, all animals were acclimatized in a pathogen-free-grade animal room under controlled conditions (21±2°C, 55%±5% humidity with a 12 h/12 h light/dark cycle) for a week and received standard laboratory chow and tap water ad libitum. All procedures for the care and handling of animals used in the study were approved by the University of Wuhan Animal Care Committee(certificate No. SYXK(E)2009-0027).

### Drugs and reagents

The following materials were obtained from commercial sources: (IMQ TCI Development Co., Ltd, Shanghai, China); dexamethasone (DXM, GrandPharma Co., Ltd, Wuhan, China); dextran sodium sulfate (DSS, molecular weight 36000–50000; MP Bio, Santa Ana, CA, USA); a myeloperoxidase (MPO) detection kit (Nanjing Jiancheng Bioengineering Institute); a fecal occult blood test kit (Baso Bio Co., LTD, Zhuhai, China); mouse TNF-α and IL-10 enzyme-linked immunosorbent assay (ELISA) kits (Bio-Swamp Life Science Lab, Wuhan, China No.MU 30030, No.MU30055) and an IL-6 ELISA kit (Elabscience Bio Co., Ltd, Wuhan, China No. M0044).

### Experimental design

The mice were randomly divided into 4 groups: control, DSS, IMQ, and DXM, with 10 mice in each group. Except in the control group, colitis was induced by giving distilled water containing 3% (w/v) DSS for 7 days. Additionally, the mice in these groups were given different drugs intraperitoneally for 7 days. The DSS group was administered sterile PBS solution, the IMQ group was given IMQ (30 mg/kg body weight) and the DXM group was treated with DXM (1.5 mg/kg body weight).

Clinical signs were observed and recorded daily during the drug-dosing period. The mice were euthanized by intraperitoneal injection of phenobarbital (160 mg/kg body weight), and the colon and spleen were dissected out. The colon length was measured, and the spleen was weighed. The colon tissues was cut into two parts. One section was placed on filter paper, pinned at the ends, fixed in 4% paraformaldehyde for hematoxylin and eosin (HE) staining, and assessed under a light microscope. The other colon segment was packaged using aluminized paper, put into liquid nitrogen, and stored at -80°C for future use.

### Evaluation of disease activity index (DAI) and histopathological score

Intestinal disease activity was assessed based on weight loss, stool consistency, and gross rectal bleeding. The overall disease severity was assessed with a clinical scoring system [17]: stool consistency (0, normal; 2, loose stool; 4, watery diarrhea); bloody stools (0, normal; 2, slight bleeding; 4, gross bleeding); and body weight loss (0, none; 1, decreased 1%-5%; 2, decreased 5–10%; 3, decreased 11%-15%; 4, decreased >15%). These values were assessed for each animal, and the sum of the 3 values constituted the DAI. A 1-cm colon segment from the anus was separated and then fixed in 4% paraformaldehyde for 24 h at room temperature. The tissue was then embedded in paraffin, stained with HE, and assessed under a light microscope. According to the criteria in the literature [18], scores were assigned for the destruction of the crypt structure, the depth of the lesions and the degree of inflammatory cell infiltration; the total of the 3 scores was recorded as the histopathological damage score.

### Assessment of MPO activity

Briefly, we weighed the colon tissue, added the appropriate amount of medium for a 5% homogenate, and processed the tissue on ice with a homogenizer until it was fully homogenized. The remaining steps were performed according to the MPO detection kit instructions, and the absorbance value was measured with a UV detector at 460 nm. The results were in U/g of tissue wet weight.

### Assay of cytokine

Cytokine levels were measured using an enzyme-linked immunosorbent assay (ELISA) method. Serum IL-6, TNF-α and IL-10 concentrations were measured with a Bio-Swamp mouse ELISA kit using monoclonal antibodies according to the manufacturer’s guidelines and expressed as ng/L.

### Statistical analysis

Statistical analyses were performed using SPSS 17.0 software. All results are expressed as the mean ± SD. The results were analyzed using GraphPad Prism version 5.0 (GraphPad Software, Inc., CA, USA). The quantitative data were tested for homogeneity of variance. Statistical comparisons between groups were made by one-way ANOVA followed by the least-significant difference (LSD) method for multiple comparisons. p<0.05 was considered statistically significant.

### Effect of IMQ on DSS-induced colitis symptoms

**Fig 1:**
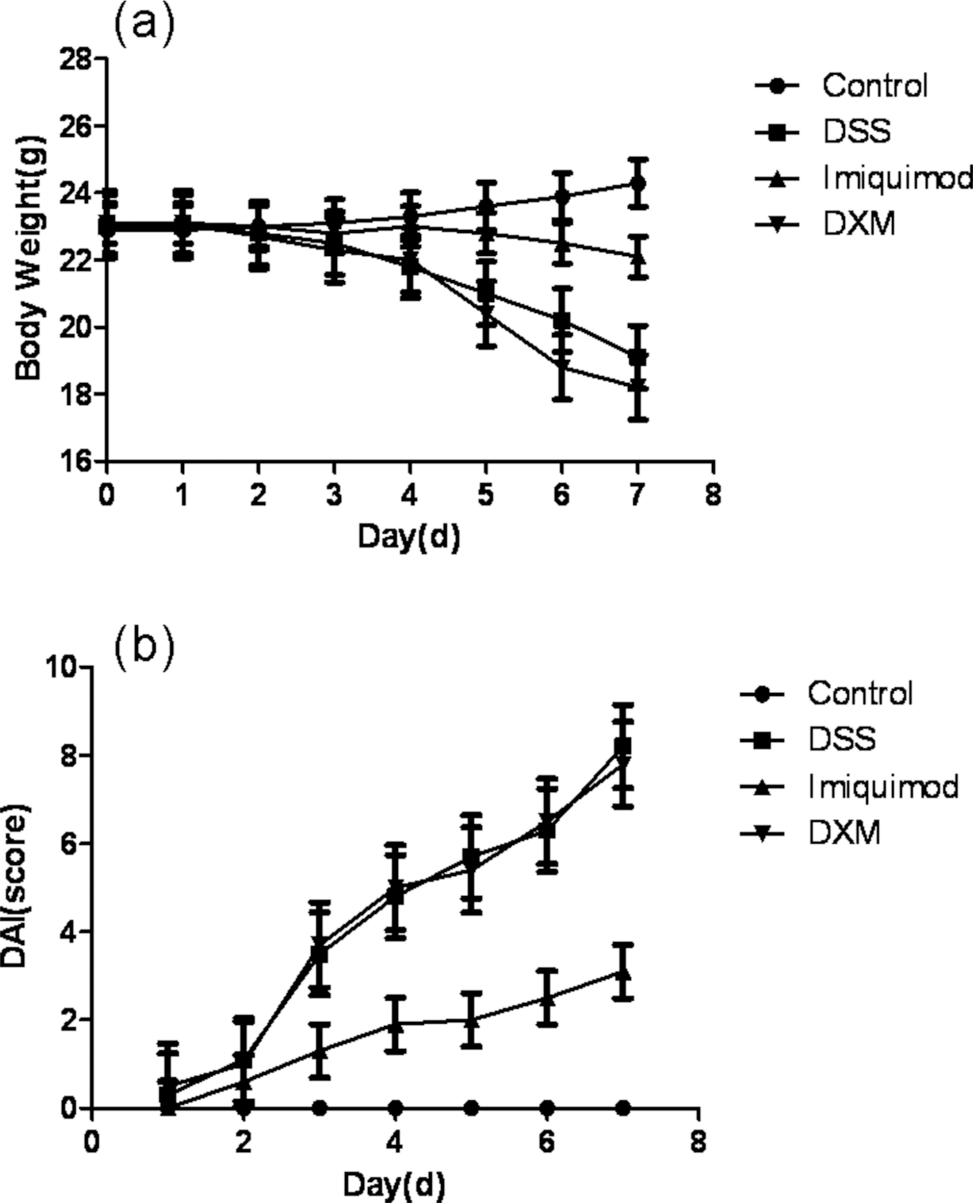
Effect of IMQ on colitis symptoms. Ulcerative colitis was induced in male C57 mice by administering 3% DSS in the drinking water for 7 days. Over the same period, IMQ and the reference compound dexamethasone were given intraperitoneally once a day. **a** Body weight was measured at the same time on each experimental day. Weight changes are given in grams (g). **b** Disease activity index scores in the four groups. Data are presented as the mean ± SD (n = 10). *P<0.05 vs control, ^#^P<0.05 vs DSS alone.

**Fig 2:**
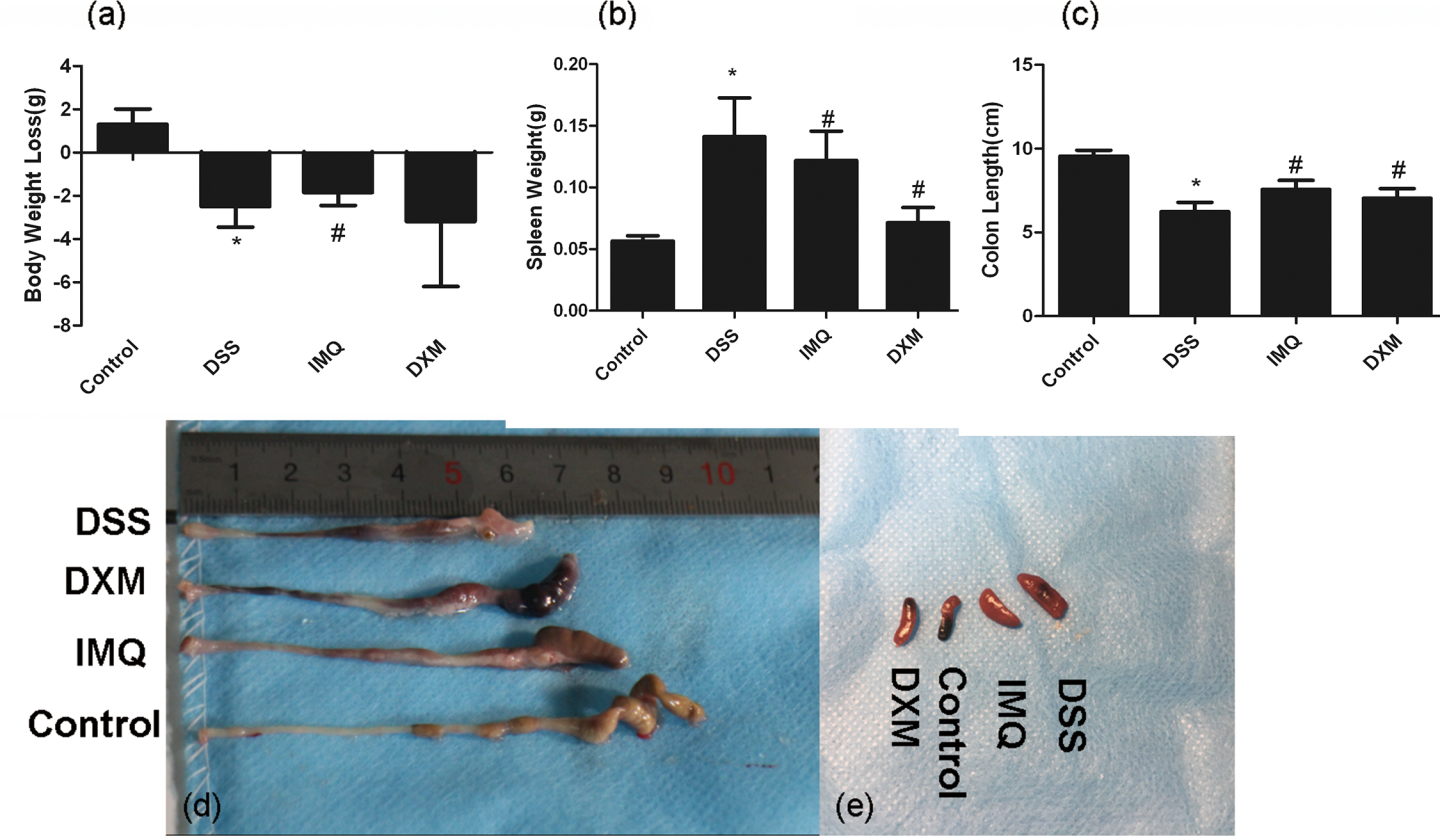
IMQ ameliorates clinical signs in DSS-induced colitis. Ulcerative colitis was induced and IMQ and DXM administered as described in the legend to Fig 1. Colon length markedly shortened in mice receiving DSS. **d**. Colons were harvested on day 7, and colon lengths were measured. **e**. Spleen weight increased in mice receiving DSS. Spleens were harvested on day 7, and spleen weight was shown. **b, c**. Colon lengths and spleen weight in the four groups. Values represent mean ± SD. *P<0.05 vs control, ^#^P<0.05 vs DSS alone.

Mice treated with 3% DSS for 7 days developed symptoms of acute colitis. DSS in conjunction with IMQ or DXM caused decreases in body weight and colon length at day 7 compared with the control group. The mean colon length and body weight of DSS-only mice showed a significant reduction compared with normal mice (P<0.05). Both IMQ and DXM alleviated the effects of DSS on body weight loss (Figs 1A and 2C) and colon length shortening (Fig 2A and 2D). DSS caused an increase in spleen weight compared with the control group, with the mean spleen weight for DSS-only mice showing a significant difference compared with normal mice (P<0.05). DSS in conjunction with IMQ or DXM also reduced the spleen weight (Fig 2B and 2E). Data of colon length and spleen weight in each group were significantly different (S1 Table).

In comparison with the control mice, the DSS-alone treatment group had a significantly increased DAI score. IMQ treatment suppressed DSS- induced colitis, ameliorated diarrhea and rectal bleeding, and reduced the loss of body weight. However, DXM treatment did not produce a significant reduction in the disease index. There was no significant difference between the DXM and DSS group (Fig 1B). The results indicated that IMQ effectively relieved DSS-induced UC symptoms (P<0.05).

### Effect of IMQ on colonic MPO activity and serum cytokine levels in DSS-induced UC in mice

**Fig 3:**
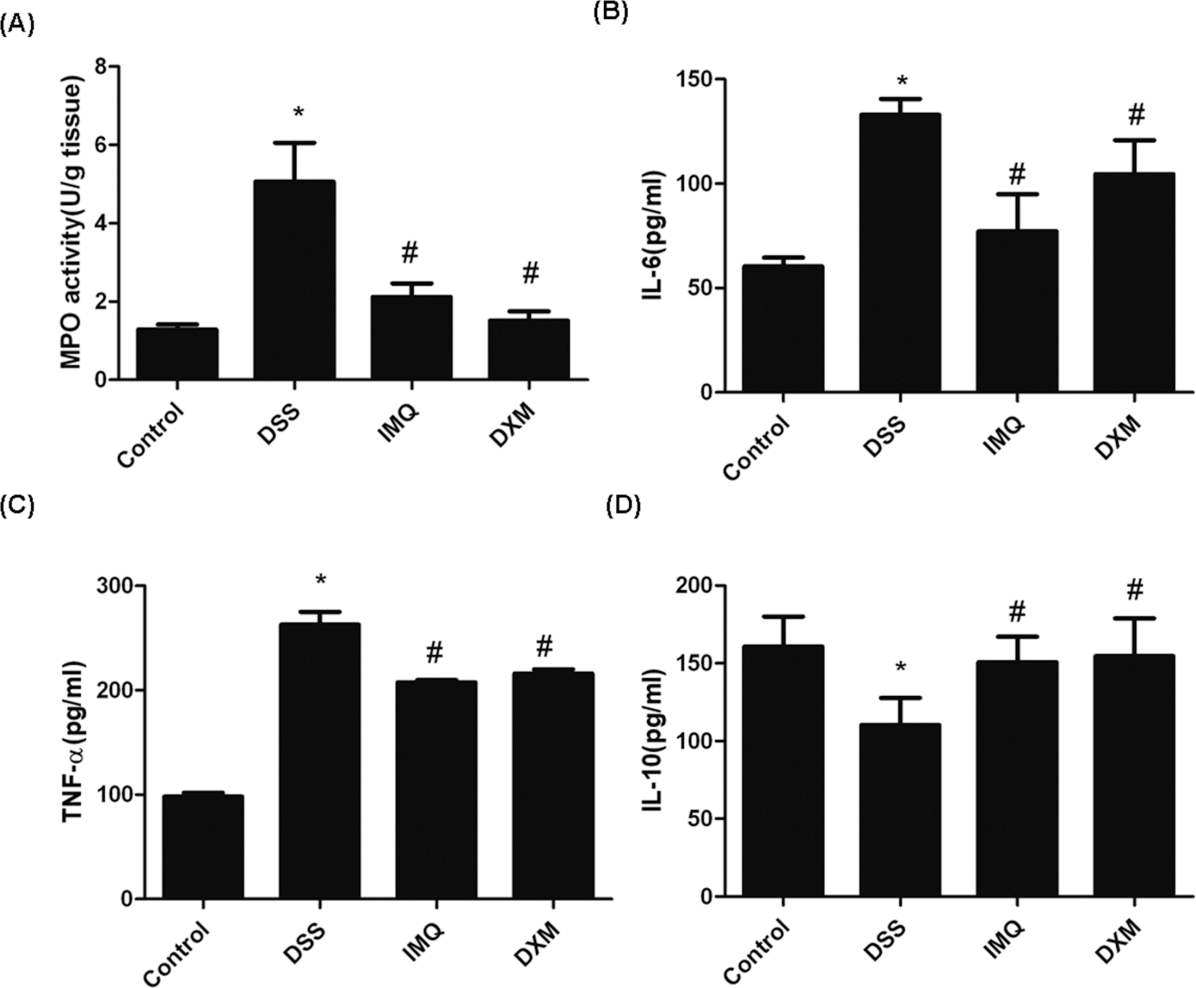
Effect of IMQ on colonic MPO activity and serum cytokine concentrations. **(A)** Colonic MPO activity was determined using an MPO detection kit. Cytokine production was determined by ELISA. **(B)** IL-6 concentration in mouse serum at day 7. **(C)** Serum TNF-α concentration in mice. **(D)** IL-10 concentration in mouse serum at day 7. Values represent mean ± SD. *P<0.05 vs control, ^#^P<0.05 vs DSS alone.

The results of increased MPO activity demonstrated a positive correlation with histopathologic and macroscopic features. The results showed a significant increase in neutrophil infiltration into the colon tissue of mice in the DSS group (Fig 3A). However, the activity of this enzyme decreased in the IMQ and DXM groups (P<0.05). Similarly, no significant difference was detected in MPO activity between the IMQ and DXM groups.

Concentration of cytokines was measured ex vivo. The serum IL-6 level was significantly higher in the DSS treatment group than in the control group (132.99 ± 7.55 pg/mL vs. 62.12 ± 6.92 pg/mL, P<0.05). IMQ and DXM showed a tendency to reduce the IL-6 level, and the reduction was statistically significant (77.18 ± 18.95 pg/mL vs. 132.99 ± 7.55 pg/mL; 104.62 ± 16.21 pg/mL vs. 132.99 ± 7.55 pg/mL, P<0.05, Fig 3B). The inflammatory TNF-α level was high in the DSS group (262.93 ± 12.14 pg/mL), but was significantly decreased in the IMQ and DXM groups; the difference was statistically significant (262.93 ± 12.14 pg/mL vs. 207.66 ± 2.31 pg/mL; 262.93 ± 12.14 pg/mL vs. 216.24 ± 3.17 pg/mL, P<0.05, Fig 3C). IL-10 expression in the DSS group was lower than that in the normal group (110.32 ± 17.36 pg/mL vs. 160.72 ± 19.30 pg/mL, P<0.05), but significantly higher in the 2 treatment groups; the difference was statistically significant (P<0.05). The mean serum IL-10 levels were 150.69 ± 16.34 pg/mL for IMQ and 154.6 6 ± 24.18 pg/mL for DXM (Fig 3D). All data between the groups of mice (S1 Fig, S2 Table).

### Intraperitoneally administered IMQ decreases histopathological damage

**Fig 4:**
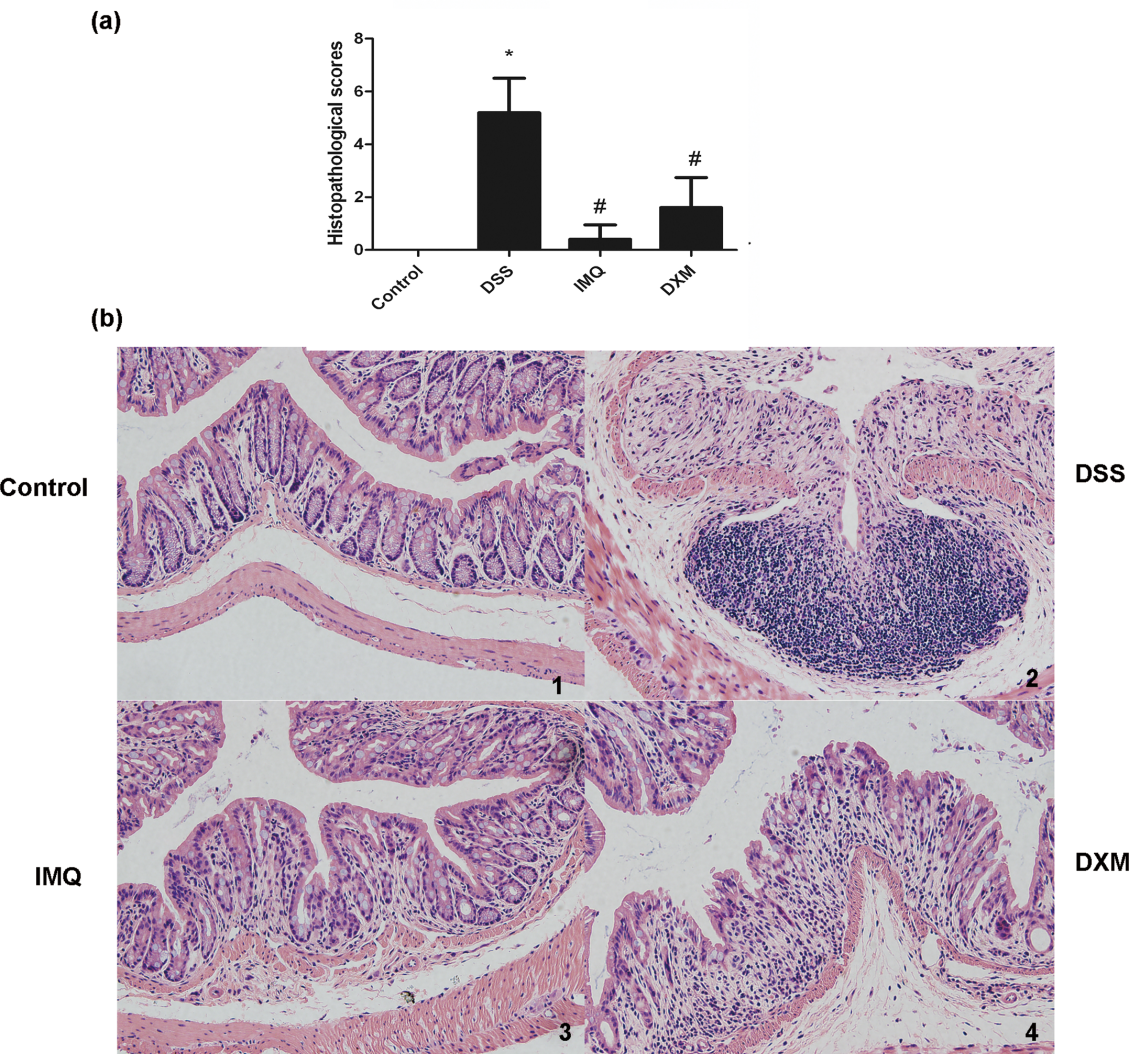
Effect of IMQ on pathological injury in colon. Ulcerative colitis was induced and IMQ and DXM administered as described in the legend to Fig 4. **a:** Pathological scores of colonic samples of each group. Data are presented as the mean ± SD (n = 6 mice). *P<0.05 vs control, ^#^P<0.05 vs DSS alone. **b:** Paraffin sections of colonic tissue were stained with hematoxylin and eosin and observed by microscope (400×). Colitis was induced by administration of 3% DSS for 7 days. 1. Control; 2. Mice treated with DSS alone; 3. Mice treated with DSS plus imiquimod; 4. Mice treated with DSS plus dexamethasone.

The colons of healthy, untreated mice showed intact epithelium and mucosa, no disruption of crypt architecture, complete goblet cells with mucus-filled vacuoles, and no infiltration of leukocytes. Severe lesions were present in DSS-treated mouse mucosa, with complete loss of colonic epithelial cells, destruction of crypt architecture, and extensive inflammatory cell infiltration; in addition, most glands were incomplete. HE staining results and histological injury scores showed that IMQ effectively protected colon tissue and attenuated DSS-induced tissue morphological changes (Fig 4A and 4B).

## Discussion

We first studied the efficacy of IMQ in a DSS-induced UC model. Mice were administered 3% DSS for 7 days to induce acute colitis and establish an acute model of UC. In the last stage of the experiment, the mice were observed to have typical UC symptoms such as weight loss, bloody mucopurulent stool and shortening of the colon. HE staining showed distinct defects in the colonic mucosa. Most of the glands were incomplete and disordered. Patients with severe lesions will suffer gland disappearance, crypt destruction, and extensive inflammatory cell infiltration. IMQ can improve the general conditions of DSS-treated mice, increase the length of the colon and the weight of the spleen, and decrease the activity of MPO in colonic tissue (Fig 2) and the expression levels of the proinflammatory cytokines TNF-α and IL-6 (Fig 3), indicating that IMQ can enhance the body’s immune response and reduce the inflammatory response. Therefore, we speculate that IMQ has a certain impact on the immune aspects of UC pathogenesis, which would further indicate that IMQ might have a certain therapeutic effect on UC inflammatory activity.

IMQ is used against a large variety of cutaneous diseases, such as genital and anal warts as a new type of immune response regulator [19], viral infections of the skin, and benign and malignant tumors. A recent study found that it can act as an immunological adjuvant [13] by regulating immune mechanisms to achieve a therapeutic effect, mainly through expression of various cytokines, which enhance the body’s inherent and acquired immune response and activate toll-like receptors (TLRs). IMQ is a TLR7 agonist and a strong inducer of type I IFN. A previous study showed that type I IFN, activated by the TLR9 signaling pathway, protects mice from experimental colitis [20].

Important studies have shown that TLR activates antigen-presenting dendritic cells (DCs) [21], which are key to CD4^+^ T-cell activation and Th1 cell differentiation. IMQ enhances the number and maturation of antigen-presenting DCs [22], increases antigen-specific CD4^+^ and CD8^+^ T- cell responses, promotes INF-γ, TNF-α, and IL-6 secretion, and induces Th1 to dominate the immune environment without suppressing the Th2 reaction; in addition, it promotes the secretion of the anti-inflammatory factor IL-10. Suzuki et al studied IMQ in the treatment of trinitrobenzene sulfonic acid-induced colitis in mice and found that IMQ significantly inhibited the inflammatory response [23], stimulated differentiation of naive T lymphocytes, and increased regulatory cells (Tregs) aggregation, thus regulating the body’s immune system. In addition, a study showed that IMQ has an inhibitory effect on respiratory inflammation in asthmatic rat models [24]. IMQ can effectively increase the expression of the transcription factor T-bet and inhibit the expression of the transcription factor GATA-3. T-bet and GATA-3 are Th1- and Th2-specific transcription factors; therefore, IMQ can regulate T lymphocyte transcription factors in two directions and then regulate the differentiation direction of Th0 cells to inhibit the Th2 cell response, thereby correcting Th1/Th2 cell imbalance and inhibiting asthmatic airway inflammation.

On the other hand, immunoregulation disorder plays an extremely important role in DSS-induced pathogenesis of UC. A study by Rutella showed that DCs play an important role in the pathogenesis of IBD [25], and that DC dysfunction can promote the development of intestinal inflammation. This may occur through activation of DCs, thereby initiating a T-cell response to pathogens, maintaining T-cell activity in inflammatory mucosa, and releasing proinflammatory cytokines [26]. Moreover, DCs recognize TLRs and selectively express TLR7 and TLR9, whereas activated TLRs trigger intrinsic and adaptive immune responses and activate NF-κB and other transcription factors, which play an important role in activating inflammatory cascades. In DSS-induced colitis, an experimental model resembling acute colitis, i.e., DC ablation during DSS administration ameliorated disease manifestation [27], suggesting that DCs are protective in the initial phases of colitis. As described in detail previously [23], DCs can efficiently stimulate naive T cells [28]; moreover, a previous report indicated that TLR9 activation induced proliferation of CCR9^+^ cells [29]. The chemokine receptor CCR9 is a key regulator of leukocyte migration and a potent inducer of Tregs. Several experiments on the intestinal mucosa of UC patients found abnormalities in Tregs and showed the effect of Th1/Th2 imbalance [30], confirming that the incidence of UC by is mediated by Th2 cell response [31]. Th2 cells can regulate atypical natural killer (NK) cells and their cytotoxicity to intestinal epithelial cells, and can induce apoptosis to increase intestinal epithelial damage. The incidence of UC is also associated with changes in cytokine levels [32, 33], with upregulation of proinflammatory cytokines (IL-6, TNF-α, IL-1β, etc.) and downregula- tion of anti-inflammatory factors (IL-4, IL-10, etc.); this persistent immune imbalance is the main cause of chronic inflammation. Given the function of IMQ as an immune adjuvant that induces a Th1-dominated immune environment and binding with TLR7 to inhibit the inflammatory response, we believe that IMQ can regulate the pathogenesis of the UC immune disorder, thereby potentially playing a role in treatment.

In our experiments, IMQ had a definite effect on DSS-induced UC, but the mechanism was unclear. A small number of studies shows that IMQ can affect the T cell response through the TLR7 signaling pathway, stimulate Treg cell aggregation, and reduce cytokine secretion, thereby inhibiting the body’s inflammatory response [21]. However, the specific mechanism needs to be further explored. In addition, a large sample of multi-center studies will be necessary to confirm the efficacy and safety of IMQ.

## Conclusions

In conclusion, our experimental studies have shown that IMQ can effectively reduce the clinical symptoms and level of inflammatory mediators in DSS-induced colitis in mice and enhance protective immunity. The results of basic scientific research imiquimod may lead to clinical applications, from the involvement of the body's own immune system to preventing the occurrence and development of UC and improving the quality of life of patients, and such research into UC biotherapy may yield future treatments with far-reaching significance. This study provides experimental evidence to show that IMQ may be a potential drug for the treatment of UC.

## Supporting information

S1 Fig Effect of IMQ on colonic MPO activity and serum cytokine levels. **(A)** Colonic MPO activity was determined using an MPO detection kit. Cytokine production was determined by enzyme-linked immunosorbent assay (ELISA). **(B)** IL-6 concentration in mouse serum at day 7. **(C)** Serum TNF-α concentration in mice. **(D)** IL-10 concentration in mouse serum at day 7. Values represent mean ± SD. *P<0.05 vs control, ^#^P<0.05 vs DSS alone.
(TIF)

S1 Table Colon length and spleen weight were significantly different among the mice. (DOC)

S2 Table Effect of IMQ on colonic MPO activity and serum IL-6, IL-10, TNF-α levels. (DOC)
