# Supplementary figures and images for: Therapeutic effect of imiquimod on dextran sulfate sodium-induced ulcerative colitis in mice
Source: PLoS One. 2017 Oct 19;12(10):e0186138. doi: 10.1371/journal.pone.0186138 (PMC5648150; doi:10.1371/journal.pone.0186138)

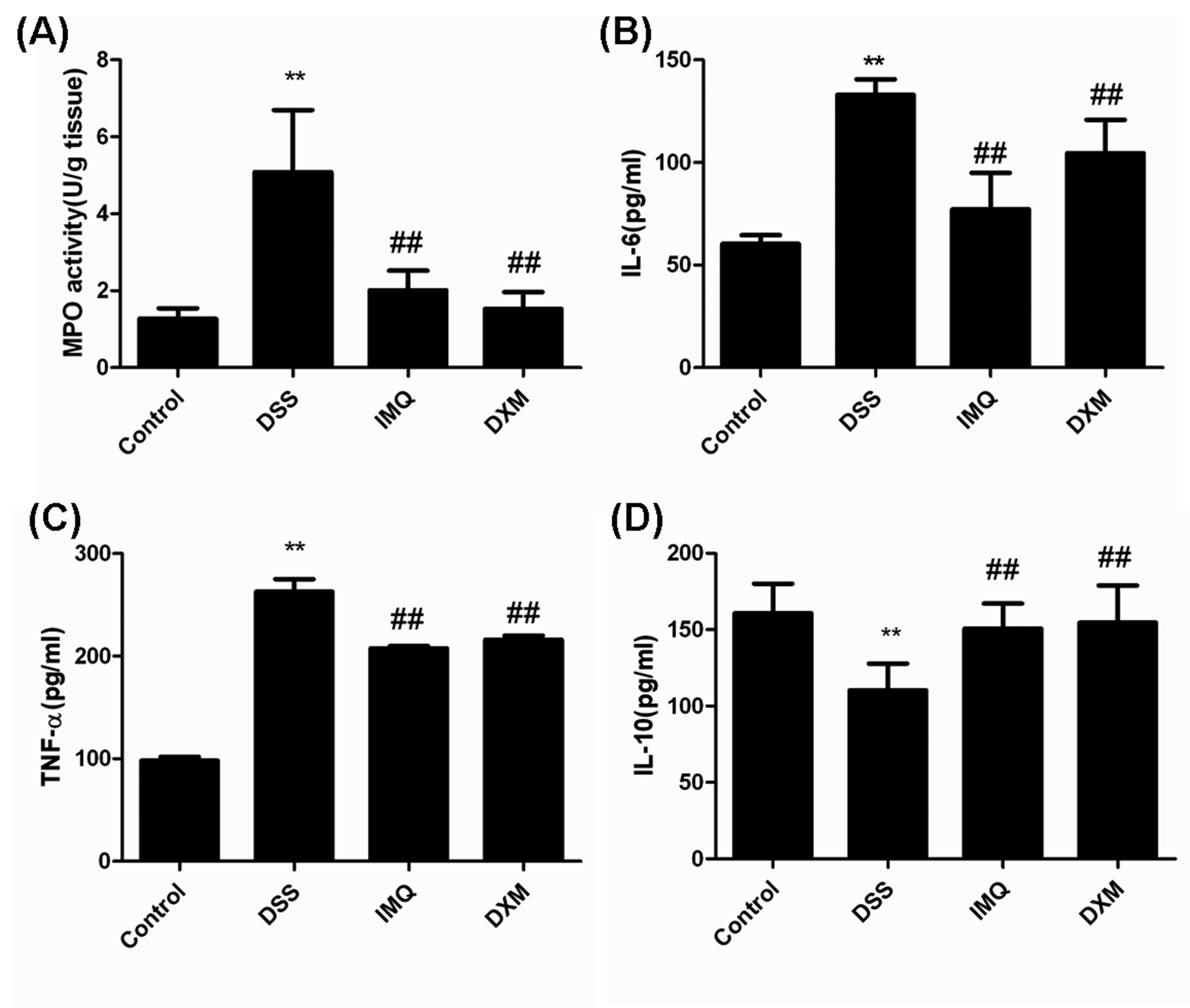

Supplement: S1 Fig — (A) Colonic MPO activity was determined using an MPO detection kit. Cytokine production was determined by enzyme-linked immunosorbent assay (ELISA). (B) IL-6 concentration in mouse serum at day 7. (C) Serum TNF-α concentration in mice. (D) IL-10 concentration in mouse serum at day 7. Values represent mean ± SD. *P<0.05 vs control, #P<0.05 vs DSS alone. (TIF) [file pone.0186138.s001.tif]
