# Supplementary material for: Therapeutic effect of imiquimod on dextran sulfate sodium-induced ulcerative colitis in mice
Source: PLoS One. 2017 Oct 19;12(10):e0186138. doi: 10.1371/journal.pone.0186138 (PMC5648150; doi:10.1371/journal.pone.0186138)
Supplement: S1 Table — (DOC) [file pone.0186138.s002.doc]

**S1 Table.** **Colon length and spleen weight were significantly different among the mice.**

| **group** | **Colon Length(cm)** | **Spleen Weight(g)** |
| --- | --- | --- |
| Conrol | 9.55 ± 0.36 | 0.056 ± 0.004 |
| DSS | 6.24 ± 0.55** | 0.141 ± 0.032** |
| IMQ | 7.57 ± 0.54# | 0.122 ± 0.024# |
| DXM | 7.04 ± 0.58 | 0.071 ± 0.012 |

The results are mean ± SD. **P<0.01 vs control, #­#P<0.01 vs DSS alone and #P<0.05 vs DSS alone.
