# Supplementary material for: Therapeutic effect of imiquimod on dextran sulfate sodium-induced ulcerative colitis in mice
Source: PLoS One. 2017 Oct 19;12(10):e0186138. doi: 10.1371/journal.pone.0186138 (PMC5648150; doi:10.1371/journal.pone.0186138)
Supplement: S2 Table — (DOC) [file pone.0186138.s003.doc]

**S2 Table. Effect of IMQ on colonic MPO activity and serum IL-6, IL-10, TNF-**α **levels.**

| **Group** | **MPO(U/g)** | **IL-6(ng/mL)** | **IL-10(ng/mL)** | **TNF-α(ng/mL)** |
| --- | --- | --- | --- | --- |
| Control | 1.283±0.136 | 62.124±6.924 | 160.715±19.298 | 98.053±3.818 |
| DSS | 5.064±0.989** | 132.990±7.545** | 110.324±17.356** | 262.925±12.139** |
| IMQ | 2.115±0.345# | 77.179±18.946## | 150.685±16.344## | 207.663±2.308## |
| DXM | 1.512±0.236 | 104.634±16.210# | 154.656±24.179## | 216.235±3.917# |

Values represent mean ± SD. **P<0.01 vs control, #­#P<0.01 vs DSS alone and #P<0.05 vs DSS alone.
